# Comparative evaluation of probe-capture and conventional metagenomic sequencing across multiple clinical sample types, with analysis of paired bronchoalveolar lavage fluid and blood samples

**DOI:** 10.1128/spectrum.04057-25

**Published:** 2026-08-04

**Authors:** Xiao Liu, Xiaoai Fan, Wanting Wu, Weiping Ni, Yingxue Hu, Qinghui Yang, Jinxing Wei, Fang Yan, Xiaoshu Chen, Jianrong Yang, Bingxue Hu, Xiaodan Yu, Wenjuan Li

**Affiliations:** 1 Department of Respiratory and Critical Care Medicine, Chengdu Fifth People’s Hospital (The Second Clinical Medical College, Affiliated Fifth People’s Hospital of Chengdu University of Traditional Chinese Medicine) 662600, Chengdu, Sichuan, Republic of China; 2 Department of Immunology and Microbiology, Zhongshan School of Medicine, Sun Yat-sen University 74644, Guangzhou, Guangdong, Republic of China; 3 Graduate school of North Sichuan Medical College 662481, Nanchong, Sichuan, Republic of China; 4 Department of Geriatrics, Geriatric Diseases Institute of Chengdu, Chengdu Fifth People’s Hospital (The Second Clinical Medical College, Affiliated Fifth People’s Hospital of Chengdu University of Traditional Chinese Medicine) 592090, Chengdu, Sichuan, Republic of China; 5 Center for Medicine Research and Translation, Chengdu Fifth People’s Hospital (The Second Clinical Medical College, Affiliated Fifth People’s Hospital of Chengdu University of Traditional Chinese Medicine) 592090, Chengdu, Sichuan, Republic of China; 6 Department of Genetics and Biomedical Informatics, Zhongshan School of Medicine, Sun Yat-sen University 74644, Guangzhou, Guangdong, Republic of China; 7 Center for Infectious Diseases, Vision Medicals Co., Ltd. 597691 , Guangzhou, Guangdong, Republic of China; 8 Department of Medical Science, Vision Medical Technology (Huzhou) Co., Ltd., Huzhou, Zhejiang, Republic of China; Nationwide Children's Hospital, Columbus, Ohio, USA

**Keywords:** probe-capture mNGS, bronchoalveolar lavage fluid, blood, cerebrospinal fluid, pathogen

## Abstract

Conventional metagenomic next-generation sequencing (mNGS) suffers from host nucleic acid interference and poor performance in low-biomass samples. Probe-capture metagenomic sequencing (PC-mNGS), which enriches microbial targets via hybridization probes, shows superior sensitivity but lacks systematic multi-sample evaluations. This study compared PC-mNGS and mNGS across diverse clinical specimens (bronchoalveolar lavage fluid [BALF], blood, cerebrospinal fluid [CSF]) and assessed the clinical utility of pathogen co-detection in paired BALF-blood samples from sepsis patients. A total of 282 samples (81 BALF, 141 blood, 25 CSF, 35 others) sequenced by both PC-mNGS and mNGS were analyzed. Additionally, 621 paired BALF-blood samples from sepsis patients with pulmonary infections were evaluated. PC-mNGS achieved higher pathogen detection rates (66.67% vs 57.10%, *P* = 0.000198) than mNGS, particularly in blood (66.67% vs 47.52%, *P* = 2.5 × 10⁻⁵). PC-mNGS detected more bacteria (19 species exclusive) and fungi (11 species exclusive) than mNGS. Viruses showed comparable detection. BALF and CSF exhibited high overall agreement (OPA: 96.30% and 88%, respectively), while blood had lower concordance (NPA: 54.05%, OPA: 70.92%). A total of 60.55% of BALF-positive samples (PC-mNGS) had co-detected pathogens in blood. Gram-negative bacteria (e.g., *Klebsiella pneumoniae*) and fungi (e.g., *Candida albicans*) showed higher blood co-detection rates than viruses. In this study, PC-mNGS detected more pathogens and showed a higher positivity rate than mNGS in blood samples. BALF sequencing data, particularly bacterial reads per million (RPM), may predict bloodstream co-detection, aiding in sepsis management. However, clinical validation and integration with traditional diagnostics are needed to confirm utility. This study highlights PC-mNGS as a promising tool for complex infections but underscores the need for rigorous multi-context validation.

**IMPORTANCE:**

Accurate and rapid identification of pathogens is critical for effective treatment of severe infectious diseases, such as sepsis. This study demonstrates that probe-capture metagenomic sequencing (PC-mNGS) detected more pathogens in blood samples compared to conventional metagenomic sequencing, especially for bacterial and fungal infections. By analyzing paired lung and blood samples, we show that high pathogen levels in lung fluid may predict bloodstream infection, offering a potential early warning for clinicians. These findings support the use of PC-mNGS as a more sensitive diagnostic tool, which could lead to faster, more targeted therapies and better outcomes for patients with complex infections.

## INTRODUCTION

Accurate pathogen identification plays an important role in infectious disease management. However, conventional diagnostic approaches continue to face persistent challenges (1, 2). Traditional culture-based methods, regarded as the gold standard, exhibit limited sensitivity due to prior antibiotic exposure and prolonged turnaround times (3–7 days) (3). Although unbiased metagenomic next-generation sequencing (mNGS) addresses some diagnostic needs through its hypothesis-free advantage in clinical applications, its utility is constrained by high host nucleic acid interference, insufficient sensitivity in low-biomass samples (e.g., 47.52% sensitivity in blood), and notable limitations in detecting co-infections or pathogens that are difficult to culture or are present at low abundance (4, 5). These challenges are particularly pronounced in samples with high host-to-microbe ratios, where clinically relevant pathogens may remain undetected.

Probe-capture metagenomic sequencing (PC-mNGS) has emerged as a revolutionary approach, utilizing pathogen-specific hybridization probes to selectively enrich microbial targets while enabling unbiased detection through non-targeted magnetic bead-based collection (3–5). Recent studies demonstrate that PC-mNGS significantly outperforms conventional methods: it elevates pathogen detection rates in blood samples to 51.6% (vs 17.4% for conventional blood culture, *P* < 0.001) (5). This technology also enables simultaneous detection of bacteria, fungi, viruses, and antimicrobial resistance genes, positioning it as a versatile diagnostic tool. Nevertheless, existing research has systematically compared PC-mNGS and mNGS only in respiratory specimens, leaving multi-sample-type evaluations unexplored. Additionally, nucleic acid-based detection methods inherently carry risks of false positives (6–9). While prior studies suggest PC-mNGS enhances pathogen detection rates in blood, the clinical validity of these findings remains uncertain, as most rely on clinical diagnoses as the gold standard without robust confirmatory evidence.

To address these gaps, we conducted a comparative study evaluating the diagnostic performance of PC-mNGS versus mNGS across diverse clinical specimens, including bronchoalveolar lavage fluid (BALF), blood, cerebrospinal fluid (CSF), and other sample types. A total of 282 samples (81 BALF, 141 blood, 25 CSF, and 35 others) were analyzed using both methods. Furthermore, we investigated 621 patients with sepsis secondary to pulmonary infections. Pathogen profiles, positive percent agreement (PPA), and negative percent agreement (NPA) were compared between methods. Co-detection rates of pathogens in paired BALF and blood samples were analyzed across different pathogen types. Receiver operating characteristic (ROC) curve analysis was employed to determine the sequence thresholds at which pathogens detected in BALF samples could be concurrently identified in blood samples. Our comprehensive evaluation highlights the enhanced diagnostic capabilities of PC-mNGS while underscoring the need for rigorous validation across varied sample types and clinical contexts. The findings aim to refine pathogen detection strategies and optimize the clinical utility of next-generation sequencing technologies.

## MATERIALS AND METHODS

### Study design

**Fig 1:**
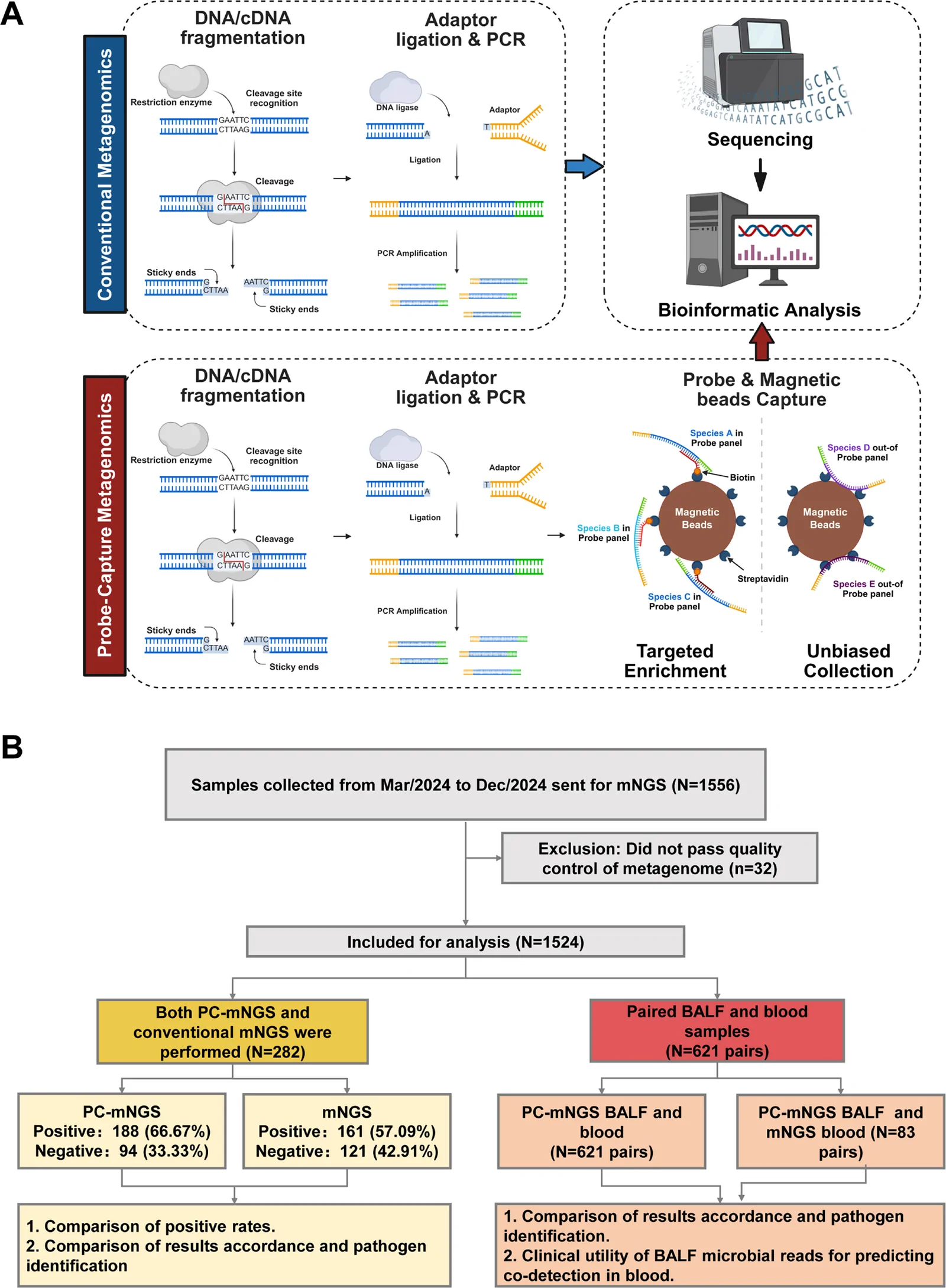
Study design. (**A**) Molecular mechanism of PC-mNGS and mNGS (reprinted from Sun, Q. et al., Clin Transl Med, 2025, under a CC BY 4.0 license). (**B**) Flowchart of sample selection and classification. In this figure, 282 denotes the number of independent specimens sequenced by both PC-mNGS and conventional mNGS (81 BALF, 141 blood, 25 CSF, 35 others). In this figure, 621 denotes 621 BALF–blood pairs from the same patients, corresponding to 1,242 individual specimens (621 × 2). For these 621 pairs, BALF was sequenced by PC-mNGS in all pairs; blood was sequenced by conventional mNGS in 83 pairs (and by PC-mNGS in the remaining pairs). Thus, the total number of individual specimens shown is 1,242 + 282 = 1,524.

We analyzed the sequenced data of 1,556 clinical samples that were referred for metagenomics sequencing between March 2024 and December 2024 from Chengdu Fifth People’s Hospital in Chengdu, China. A total of 282 samples were sequenced using both conventional mNGS and PC-mNGS. Additionally, a total of 621 pairs of matched BALF and blood samples obtained from the same patient on the same day and shown to be pathogen positive in BALF by PC-mNGS were further examined to compare and assess the detection capabilities of blood and BALF metagenomics tests for pathogens. Among the 621 pairs, all were sequenced by PC-mNGS, and 83 blood samples also had mNGS data (Fig. 1). This study was conducted in accordance with the Declaration of Helsinki. Study protocols were reviewed and approved by the Ethics Committee of Chengdu Fifth People’s Hospital (approval number: Ethics Review 2023-001(Dept)-01). The requirement for written informed consent was waived given that this was a retrospective analysis using clinical specimens and that no identifying patient information was involved. Samples were referred for metagenomic sequencing at the discretion of treating clinicians based on clinical suspicion of infection and were not pre-selected according to culture status. The cohort therefore included both culture-positive and culture-negative specimens.

### Metagenomics sequencing

### Host depletion, nucleic acid extraction, library construction, probe-capture hybridization, and sequencing

For both conventional mNGS and PC-mNGS, the operational process can be summarized into the following steps (10): (i) DNA extraction; (ii) RNA extraction and reverse transcription; (iii) library construction; (iv) probe hybridization capture (optional for probe-capture metagenomics); and (v) sequencing. For samples tested by both methods, aliquots from the same clinical specimen were processed in parallel. Nucleic acids were extracted using the same pretreatment and extraction workflow, and libraries were prepared using the same protocol. The PC-mNGS workflow included an additional probe hybridization capture step after library construction, whereas the conventional mNGS libraries were sequenced directly. (i) For whole blood, centrifugation was applied to separate cellular components: 1,600 × *g* for 10 min to pellet host cells, followed by collection of the supernatant for downstream processing. BALF was pre-treated with trypsin (final concentration 1%). CSF was gently shaken to mix the sample for later use. Next, 1 U of Benzonase (Sigma) and 0.5% Tween 20 (Sigma) were used to deplete the host nucleic acid in 50 μL of precipitate, which was then incubated for 5 min at 37°C. The reaction was stopped by adding 400 μL of terminal buffer. The QIAamp UCP Pathogen Mini Kit was then used to extract and elute the nucleic acid from 400 μL of pretreatment samples in 60 μL of elution buffer (catalog number: 50214; Qiagen, Germany). (ii) Using the QIAamp Viral RNA Mini Kit (catalog number: 52904; Qiagen, Germany), 200 μL of the mixture was extracted in accordance with the manufacturer’s instructions for RNA extraction and reverse transcription. Using the QIAseq FastSelect-rRNA HMR Kit (catalog number: 334386; Qiagen, Germany), ribosomal RNA removal was performed on total RNA prior to reverse transcription. cDNA was then generated using reverse transcriptase and dNTPs (Thermo Fisher Scientific, USA); a Qubit double-stranded DNA (dsDNA) high-sensitivity (HS) assay kit (catalog number: Q32854; Invitrogen, USA) was used to quantify both DNA and cDNA (11, 12). (iii) Following the manufacturer’s instructions, the indexed DNA/cDNA libraries were then created using a KAPA low throughput library creation kit (KAPA Biosystems, U.S.A.). (iv) For hybrid capture-based microbial probe enrichment in one hybridization cycle, a 750-ng aliquot of each sample was utilized (SeqCap EZ Library, Roche, U.S.A.). The HUBDesign pipeline (13) was utilized to design the probe, and the probes were designed as previously described (5). Pathogens used for probe design are listed in Table S1. Briefly, probes were designed using the HUBDesign pipeline with default parameters. This pipeline leverages the mosaic genomic composition of pathogens to generate probes from representative sequences across family, genus, and species taxonomic levels, enabling clade-specific enrichment and detection of both known and novel microorganisms. The approach has been validated for comprehensive detection of all sequenced coronaviruses and sepsis-associated bacterial pathogens (13). The probe-capture assay used in this study is covered by a China National Intellectual Property Administration invention patent (Patent No. ZL2020116367800). (v) The library concentration was determined using a Qubit dsDNA HS Assay Kit. An Agilent 2100 Bioanalyzer (Agilent Technologies, Santa Clara, CA, USA) and a High Sensitivity DNA kit were used to evaluate the quality of the library. An Illumina NextSeq 550 sequencer (Illumina, U.S.A.) was used to sequence the library using a 75-cycle single-end sequencing approach.

### Bioinformatic analysis

Reads less than 40 bp, low-quality reads, duplicate reads, and adapter contamination were eliminated using Trimmomatic (v0.39) (14). The default parameters of fastp (v0.23.4) eliminated low-complexity reads (15). By using Burrows-Wheeler Aligner software (BWA-0.7.17) (16) to compare the human sequence data to the hg38 reference genome, the human sequence data were found and removed. Pathogens and their genomes or assemblies were chosen from the NCBI Assembly and Genome databases (https://benlangmead.github.io/aws-indexes/k2) in accordance with the Kraken 2 (v2.1.3) criteria for choosing representative assemblies for microorganisms (bacteria, viruses, fungi, protozoa, and other multicellular eukaryotic pathogens) in order to create the microbial genome database. Utilizing BWA software, microbial reads were matched to the database (16). Mapped reads were defined as those that have 90% identity to the reference (7). Additionally, the secondary analysis did not include reads that had numerous locus alignments within the same genus. Only reads that were mapped to the genome of the same species were considered (7).

We normalized the sequencing reads using reads per million (RPM) to account for variations in sequencing depth across samples. Samples spiked with microorganisms were classified as positive samples, and the negative control was defined as the negative sample. For species with cultured isolates, positive cutoff values were determined by ROC analysis, with the RPM corresponding to the highest area under the curve (AUC) selected as the species-specific threshold. For microorganisms without cultured isolates, the mean RPM and standard deviation were calculated, and the mean RPM + 3 SD was set as a positive cutoff value (17).

In accordance with the three references listed in a prior study, the clinical reportable range for pathogens was determined (17): (i) Johns Hopkins ABX Guide (https://www.hopkinsguides.com/hopkins/index/Johns_Hopkins_ABX_Guide/Pathogens); (ii) Manual of Clinical Microbiology; and (iii) clinical case reports or research articles published in peer-reviewed journals. All clinical reportable pathogens are listed in Table S2.

### Quality control

Sterile deionized water was generated as non-template controls to track the possible sources of contamination. Both were prepared in parallel with other samples in each batch (18, 19). To create the background microbe list in our lab, we also utilized sterile cotton swabs dipped in sterile deionized water to wash off the centrifuge and biosafety cabinet surfaces.

### Statistical analysis

The chi-square test was used to compare differences in categorical variables, while the Mann-Whitney U test was employed for continuous variables using SPSS version 23.0 (SPSS, Inc., Chicago, IL, USA). GraphPad Prism 6.0 (GraphPad software) and R (R Foundation for Statistical Computing, Vienna, Austria) were used for drawing figures. Statistical significance was present when *P* < 0.05.

## RESULTS

### PC-mNGS versus mNGS in pathogens’ detection

Among all included samples, 282 were sequenced by both PC-mNGS and mNGS (Fig. 1A), including 81 BALF, 141 blood, 25 CSF, and 35 other sample types (tissue, sputum, drainage liquid, ascites, bile, pus, and pleural effusion; summary of samples and detected species are listed in Table S3). Figure 1B illustrates the molecular mechanism of PC-mNGS. PC-mNGS generated a significantly higher median microbial read count of 658,599 reads per sample (interquartile range [IQR]: 193,186–1,552,262) compared with 97,394 reads per sample (IQR: 20,066–1,724,461) for conventional mNGS, corresponding to a 6.76-fold enrichment (Wilcoxon signed-rank test, *P* = 6.5e-5, Fig. S1A). These findings demonstrate that the probe-capture enrichment strategy consistently and substantially increases the proportion of microbial sequences recovered, particularly in sample matrices characterized by high host nucleic acid content, such as blood, where the analytical sensitivity of conventional mNGS is inherently constrained.

**Fig 2:**
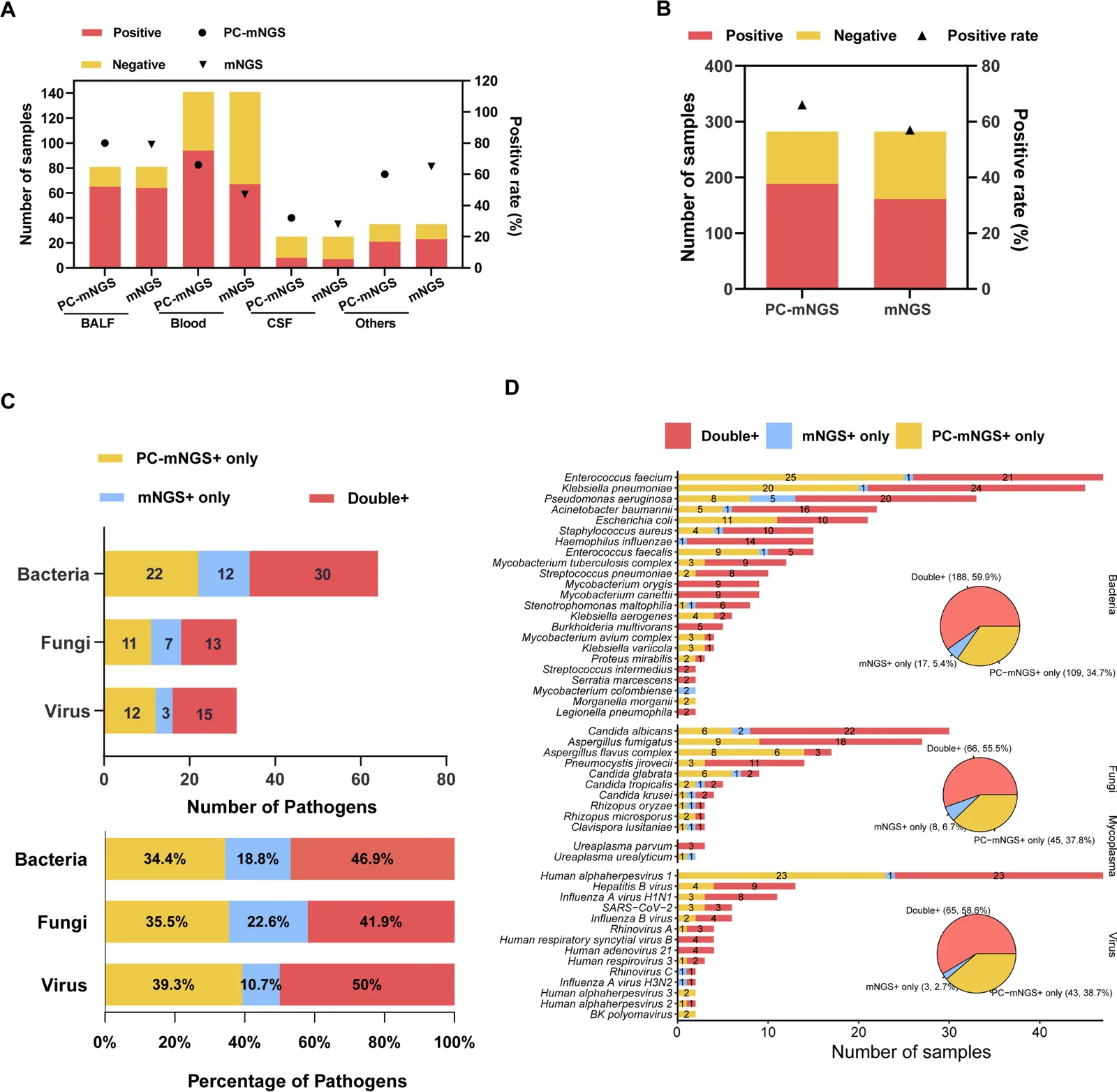
Comparison of PC-mNGS and mNGS in pathogens’ detection among different sample types. (**A and B**) Number of positive and negative samples and corresponding positive rates of two methods among different sample types (**A**) and total samples (**B and C**) The number of bacterial, fungal, and viral pathogens at the species level that were detected by PC-mNGS only, mNGS only, or both. The percentages shown below the panels indicate the corresponding proportions of detected species within each pathogen category. (**D**) The number of samples that were shown to be positive for the corresponding pathogens at the species level by PC-mNGS only, mNGS only, or both (only those with a sample number of more than 1 are shown). The pie chart indicates the corresponding proportions (%) of bacteria, fungi, and virus detections across PC-mNGS only, mNGS only, and both.

The positive rates of PC-mNGS for BALF, blood, and CSF were 80.25%, 66.67%, and 32%, respectively. Whereas mNGS showed a positive rate of 79.01%, 47.52%, and 28% among BALF, blood, and CSF samples (Fig. 2A). The results revealed that PC-mNGS obtained a significantly higher positive rate than mNGS among blood samples (*P =* 0.000025). For all samples, PC-mNGS exhibited an overall microbial positive rate of 66.67% (188/282), significantly higher than that of mNGS, which was 57.10% (161/282, *P* = 0.000198, Fig. 2B). By comparing the results’ consistency between the two assays, we demonstrated the PPA, NPA, and OPA between PC-mNGS and mNGS in Table 1. The OPA was 96.30%, 70.92%, and 88% for BALF, blood, and CSF samples, respectively. Among all 282 samples, they obtained an OPA of 81.91%.

**TABLE 1:** Results consistency between PC-mNGS and mNGS among different sample types^*a*^

Sample type	PC-mNGS	mNGS		PPA%	NPA%	OPA%
		Positive	Negative			
BALF	Positive	63	2	98.44	88.24	96.30
	Negative	1	15			
Blood	Positive	60	34	89.55	54.05	70.92
	Negative	7	40			
CSF	Positive	6	2	85.71	88.89	88.00
	Negative	1	16			
Others	Positive	20	1	86.96	91.67	88.57
	Negative	3	11			
Total	Positive	149	39	92.55	67.77	81.91
	Negative	12	82			

^*a*^ BALF, bronchoalveolar lavage fluid; CSF, cerebrospinal fluid; PPA, positive percent agreement; NPA, negative percent agreement; OPA, overall percent agreement; PC-mNGS, probe-capture metagenomic next-generation sequencing.

As for the comparison of detected pathogens between the two methods (Fig. 2C), a total of 30 bacteria (46.9%), 13 fungi (41.9%), and 15 viruses (50%) were co-detected by PC-mNGS and mNGS. However, there were 12 bacteria (18.8%), 7 fungi (22.6%), and 4 viruses (10.7%) that were only detected by mNGS; many more kinds of pathogens, including 22 bacteria (34.4%), 11 fungi (35.5%), and 12 viruses (39.3%), were detected by PC-mNGS only, within the predefined clinical reportable range and pathogen-calling framework, and without independent confirmation (Fig. 2C). Figure 2D illustrated the positive sample numbers of specific pathogens by two assays. *Klebsiella pneumoniae*, *Enterococcus faecium (E. faecium)*, *Pseudomonas aeruginosa*, and *Haemophilus influenzae* were the most frequently detected bacteria in both PC-mNGS and mNGS assays. *Acinetobacter baumannii* and *Staphylococcus aureus* were also the bacteria that were detected at a high frequency. For fungi detection, *Candida albicans* was the most frequently detected species in both assays, followed by *Aspergillus fumigatus* and *Pneumocystis jirovecii*. In terms of viral detection, human alphaherpesvirus 1 (HSV-1, human herpes simplex virus 1) showed a remarkable detection frequency, followed by influenza A virus H1N1 and hepatitis B virus. Collectively, it could be observed that the largest fraction of positive samples was detected by both methods (59.9% for bacteria, 55.5% for fungi, and 58.6% for viruses), followed by PC-mNGS positivity only (34.7% for bacteria, 37.8% for fungi, and 38.7% for viruses). These results indicate that PC-mNGS detected substantially more pathogens than mNGS (Fig. 2D).

Among co-detected sample-pathogen pairs, PC-mNGS generally yielded higher pathogen read counts than conventional mNGS. At the category level (Fig. S1B), PC-mNGS showed significantly higher read counts for bacteria (median, 1,987.5 vs 379 reads; *P* = 3.3e-9) and fungi (median, 407 vs 85 reads; *P* = 0.003). At the species level (Fig. S1C), PC-mNGS produced significantly higher read counts for several common co-detected pathogens, including *K. pneumoniae* (*P* = 5.0e-4), *E. faecium* (*P* = 9.9e-5), *P. aeruginosa* (*P* = 0.004), *A. fumigatus* (*P* = 4.8e-4), *A. baumannii* (*P* = 0.024), and human alphaherpesvirus 1 (*P* = 5.8e-4). These findings indicate that PC-mNGS improves read recovery for many co-detected bacterial and fungal pathogens, while the gain is pathogen-dependent.

### PC-mNGS versus mNGS in pathogen detection among blood samples

**Fig 3:**
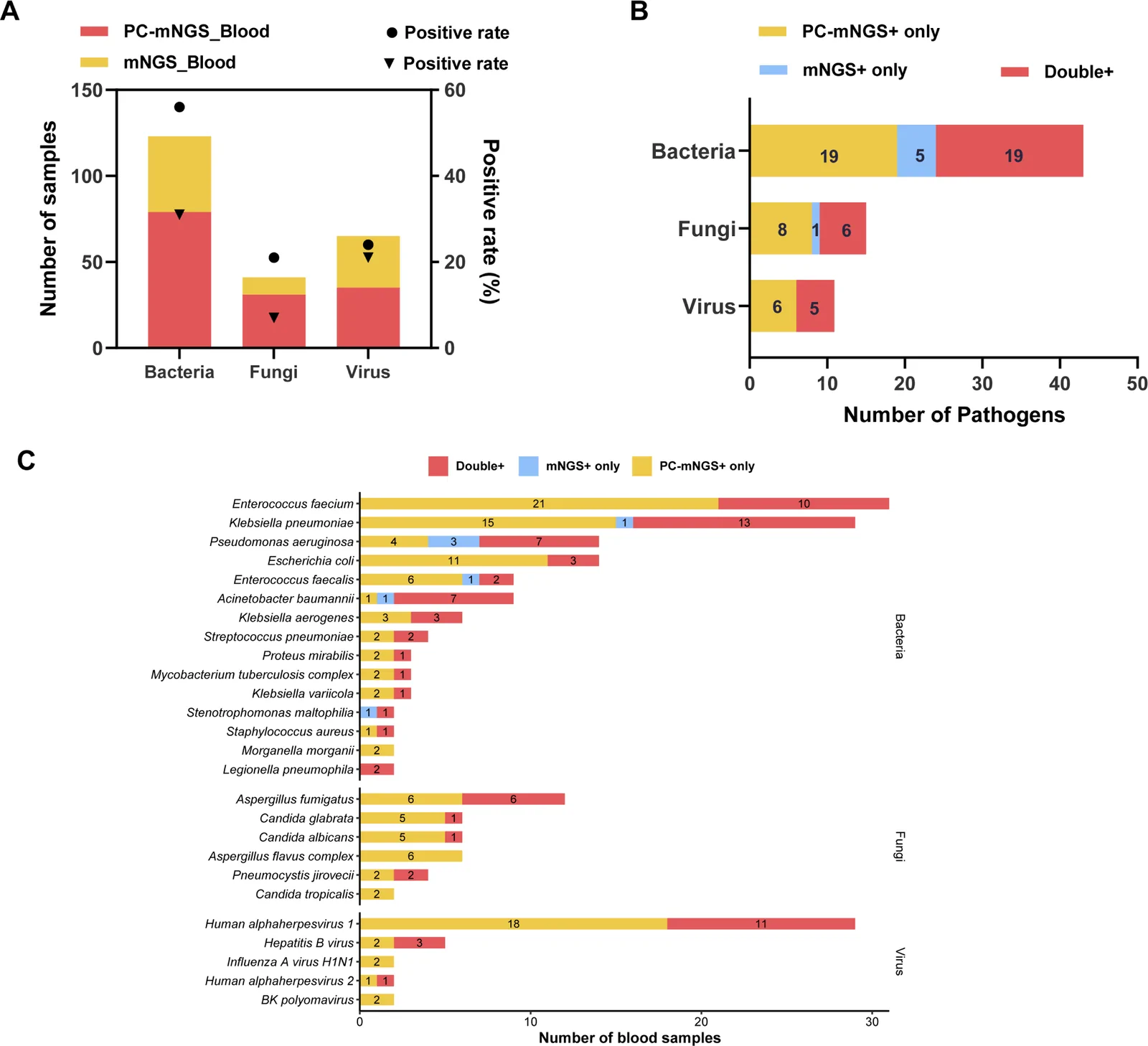
Comparison of PC-mNGS and mNGS in pathogens’ detection among blood samples. (**A**) Number of positive and negative samples and corresponding positive rates of two methods among blood samples for bacteria, fungi, and viruses. (**B**) The number of bacterial, fungal, and viral pathogens at the species level that were detected by PC-mNGS only, mNGS only, or both among blood. (**C**) The number of samples that were shown to be positive for corresponding pathogens at the species level by PC-mNGS only, mNGS only, or both among blood (only those with a sample number of more than 1 are shown).

We observed that PC-mNGS showed a significantly higher positive rate than mNGS among blood samples (*P =* 0.000025, Fig. 2A), and their NPA was relatively low (54.05%, Table 1). To disclose the underlying differences, we investigated their pathogen consistency among 141 blood samples. As illustrated in Fig. 3A, PC-mNGS achieved a positive rate of 56.03% (79/141) for bacterial detection, significantly higher than mNGS, which was 31.21% (44/141, *P =* 0.000026). For fungal detection, there were 31 (21.99%) and 10 (7.09%) samples shown to be fungi positive by PC-mNGS and mNGS, respectively, with a significant difference among detection rates (*P =* 0.000389). A comparable number of samples (35 vs 30) were shown to be virus positive by both methods. In terms of the number of pathogens, the two assays detected in blood (Fig. 3B), there were 19 kinds of bacteria and 8 species of fungi only detected by PC-mNGS. *E. faecium, Klebsiella pneumoniae,* and *Aspergillus fumigatus* were the most frequently detected bacteria and fungi in blood by both assays (Fig. 3C). Also, a portion of viruses was only detected by PC-mNGS (Fig. 3C). It could be observed that PC-mNGS identified an obviously increased number of pathogens, especially for bacteria (Fig. 3).

### Pathogen concordance of BALF and paired blood samples in mNGS assay

**Fig 4:**
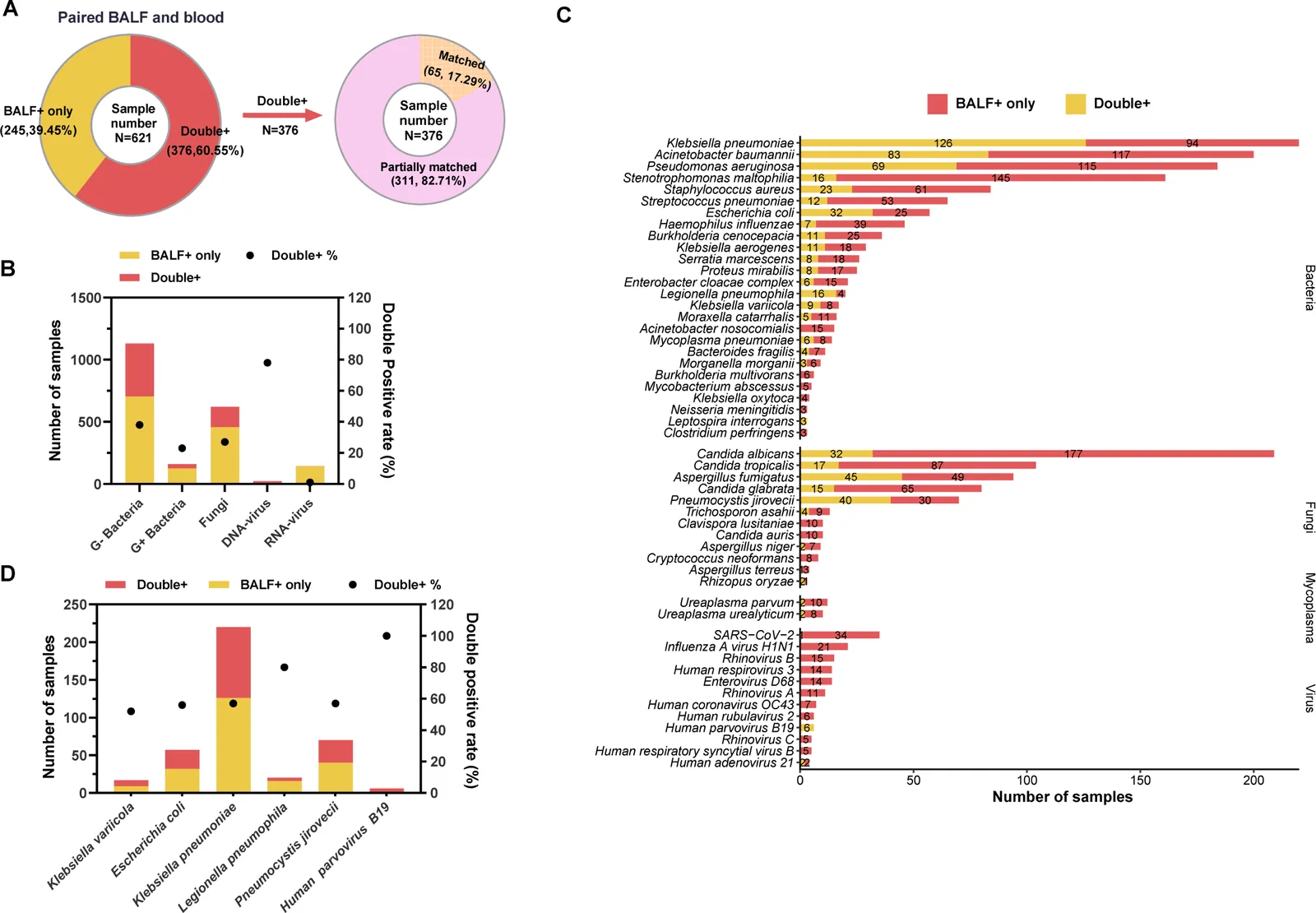
Analysis of pathogen concordance of BALF samples and paired blood samples using PC-mNGS. (**A**) Pie chart demonstrating the results’ consistency of PC-mNGS between blood and paired BALF samples. The double-positive samples were further categorized as matched (detected pathogens were identical) and partially matched (at least one overlap of pathogens was observed). “Matched” indicates that the exact same pathogen species were detected in both BALF and blood; “Partially matched” indicates that at least one pathogen species was shared but was not identical between the two samples. (**B**) The number of bacterial, fungal, and viral pathogens at the species level that were detected in BALF only, or both BALF and blood, and the corresponding double-positive rate using PC-mNGS. (**C**) The number of samples that were shown to be positive for corresponding pathogens at the species level in BALF only, or both BALF and blood, by PC-mNGS (only those with a sample number of more than 2 are shown). (**D**) The prominent pathogens that could be co-detected in BALF and paired blood using PC-mNGS.

Furthermore, we analyzed 621 pairs of BALF and blood samples that were sequenced by PC-mNGS. Among these samples, 245 pairs (39.45%) were shown to be pathogen positive in BALF samples only, and the remaining 376 pairs (60.55%) showed pathogen positivity in both BALF and paired blood samples (Fig. 4A). Importantly, for diagnostic development, this paired analysis addresses whether the pathogen signal is present in BALF only or shared across both compartments.

To better reflect clinical relevance, we further classified the double-positive pairs based on pathogen concordance. Specifically, 65 pairs (17.29%) were matched, indicating that the detected pathogen species were identical between BALF and blood, while the remaining 311 pairs (82.71%) were partially matched, indicating that both compartments were positive but the detected pathogen species composition was not identical (i.e., only partial overlap was observed) (Fig. 4A). Consistently, several pathogens were identified only in BALF, especially for RNA viruses, supporting that BALF can reveal compartment-restricted pathogens that may not be captured in blood (Fig. 4B). Compared with Gram-positive (G+) bacteria, Gram-negative (G−) bacteria were more likely to be co-detected in blood (37.8% vs 23.0%, *P =* 0.000237, Fig. 4B). The dominating pathogens detectable in BALF and paired blood by PC-mNGS included *Klebsiella pneumoniae*, *Acinetobacter baumannii*, and *Pseudomonas aeruginosa*, *Candida albicans*, *Aspergillus fumigatus*, and *Pneumocystis jirovecii*. In contrast, most viruses were observed predominantly in BALF (Fig. 4C). Notably, more than half of *Klebsiella pneumoniae*, *Pneumocystis jirovecii*, and *Escherichia coli* were observed across both compartments (Fig. 4D). These patterns suggest that detection of high-abundance bacterial or fungal pathogens in BALF frequently correlates with bloodstream involvement or circulation of microbial nucleic acids, whereas viral detections are largely compartmentalized to the lung.

**Fig 5:**
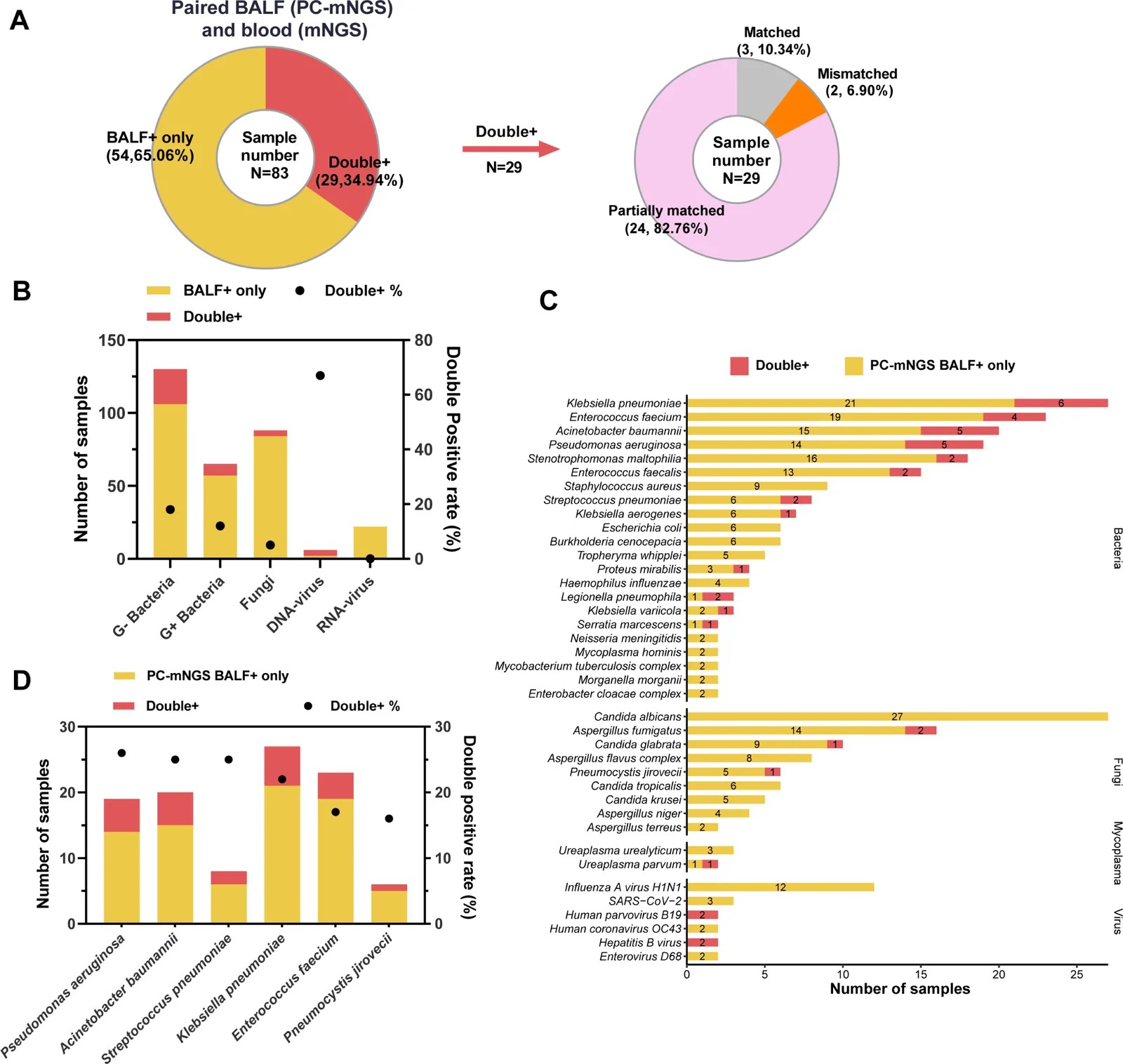
Analysis of pathogen concordance of BALF samples and paired blood samples using PC-mNGS and paired blood samples using mNGS. (**A**) Pie chart demonstrating the results’ consistency of PC-mNGS BALF between paired mNGS blood samples. The double-positive samples were further categorized as matched (detected pathogens were identical) and partially matched (at least one overlap of pathogens was observed). “Matched” indicates that the exact same pathogen species were detected in both BALF and blood; “Partially matched” indicates that at least one pathogen species was shared but was not identical between the two samples. (**B**) The number of bacterial, fungal, and viral pathogens at the species level that were detected in BALF only, or both BALF and blood, and the corresponding double-positive rate. (**C**) The number of samples that were shown to be positive for the corresponding pathogens at the species level in PC-mNGS BALF only, or both BALF and mNGS blood (only those with a sample number of more than 1 are shown). (**D**) The prominent pathogens that could be co-detected in BALF by PC-mNGS and paired blood using mNGS.

Additionally, 83 blood samples also had metagenomic data generated by conventional mNGS, allowing comparison of the pathogen spectrum between BALF detected by PC-mNGS and paired blood detected by mNGS (Fig. 5). Among these 83 pairs, the majority (54/83, 65.06%) exhibited BALF-positive only, while the remaining 29 pairs were double positive in both sample types (Fig. 5A). Within the double-positive cohort, 24/29 (82.76%) showed partial concordance between BALF and blood, consistent with the observation that signals are often present in both compartments but are not compositionally identical (Fig. 5A).

Across pathogen categories, G− bacteria demonstrated a relatively higher double-positive concordance than G+ bacteria (18.5% vs 12.3%, *P* > 0.05, Fig. 5B). At the species level, BALF samples detected by PC-mNGS exhibited superior pathogen detection capacity, with only a limited number of predominantly bacterial pathogens, such as *Pseudomonas aeruginosa*, *Streptococcus pneumoniae*, and *Acinetobacter baumannii*, being co-detected in blood specimens by mNGS (Fig. 5C and D). Collectively, these findings support that conventional mNGS demonstrates inferior pathogen detection performance compared with probe-capture enhanced metagenomic sequencing in paired BALF–blood assessment.

### Clinical utility of BALF microbial reads for predicting co-detection in blood

**Fig 6:**
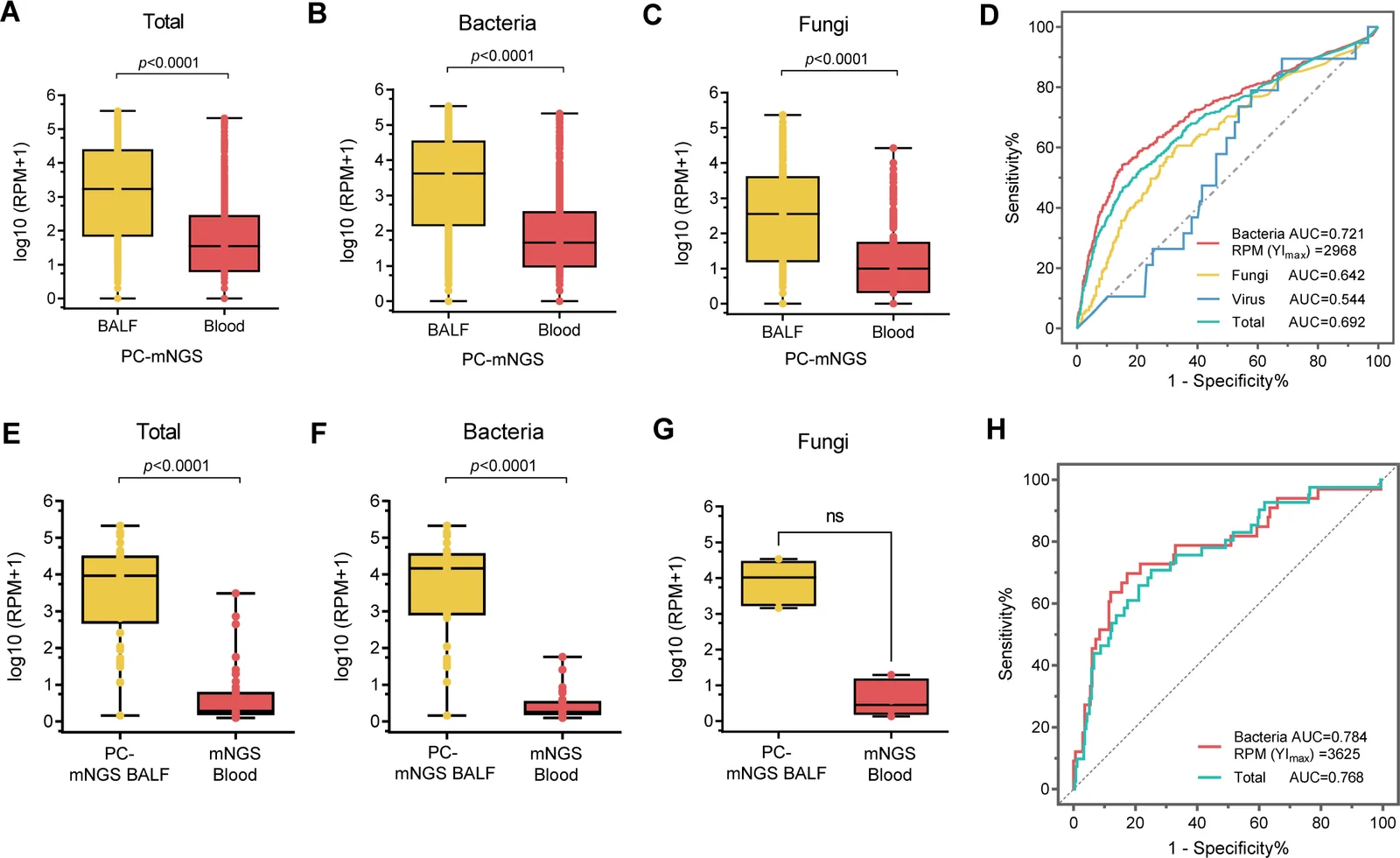
Clinical utility of BALF microbial reads for predicting co-detection in blood. Analyses were restricted to co-detected pathogen species (the same species positively called in both BALF and paired blood from the same patient). (**A–C**) Comparisons of reads number (reads per million, RPM) of total pathogens (**A**), bacterial pathogens (**B**), and fungal pathogens (**C**) between BALF and blood using PC-mNGS for their co-detected pathogens. (**D**) The ROC curve for PC-mNGS BALF microbial RPM in predicting paired blood co-detection. (**E–G**) Comparisons of reads number (reads per million, RPM) of total pathogens (**A**), bacterial pathogens (**B**), and fungal pathogens (**C**) between BALF using PC-mNGS and blood using mNGS for their co-detected pathogens. (**H**) The ROC for PC-mNGS BALF microbial RPM in predicting paired blood co-detection using mNGS. For ROC analysis, the microorganisms at species level detected in BALF were assigned to blood co-detected and blood negative groups. The cutoff value for RPM was equal to the RPM with the YI_max_ (Youden index maximum).

**Fig 7:**
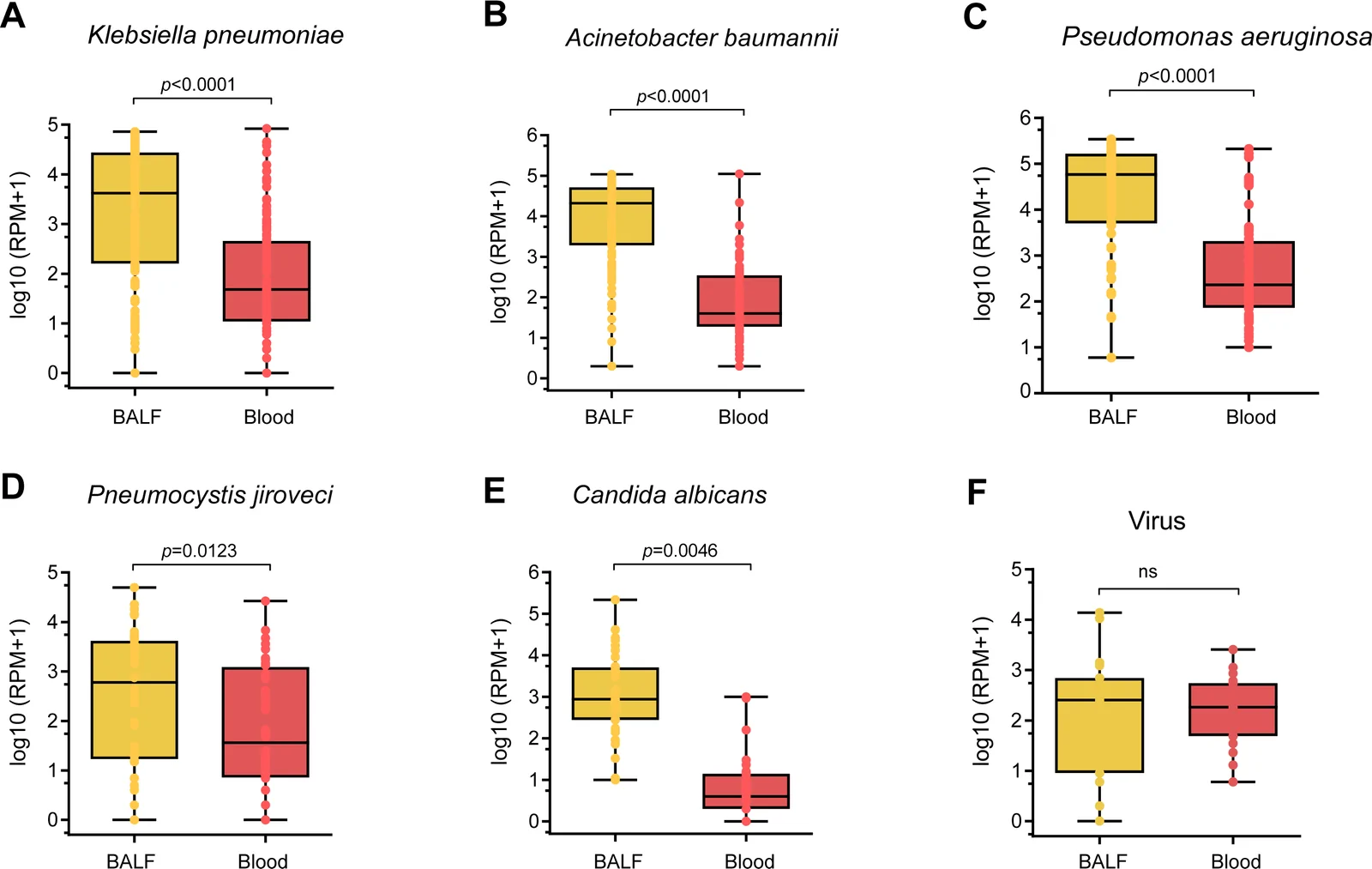
Comparisons of reads number (reads per million, RPM) of illustrated pathogens, including *Klebsiella pneumoniae* (**A**), *Acinetobacter baumannii* (**B**), *Pseudomonas aeruginosa* (**C**), *Pneumocystis jirovecii* (**D**), *Candida albicans* (**E**), and viral pathogens (**F**), between BALF and blood using probe-capture mNGS for their co-detected pathogens. Owing to the small number of double-positive viral pairs (*n* = 19), viruses were analyzed as a combined group; no significant difference was observed between BALF and blood.

We subsequently compared the microbial reads number of co-detected pathogens between BALF and paired blood. It was demonstrated that BALF samples had significantly more reads of total, bacterial, and fungal pathogens than blood detected by PC-mNGS (*P <* 0.0001, Fig. 6A through C). In terms of specific pathogens detected by PC-mNGS, including *Klebsiella pneumoniae*, *Acinetobacter baumannii*, *Pseudomonas aeruginosa*, *Pneumocystis jirovecii*, and *Candida albicans*, their reads number was also markedly higher in BALF than in blood (*P <* 0.05, Fig. 7A through E). Meanwhile, the reads number of viral pathogens was comparable between BALF and blood (*P =* 0.860, Fig. 7F). As for the comparison of reads number between PC-mNGS detected BALF and mNGS detected blood, BALF also exerted remarkably more reads of total pathogens, bacteria, and fungi than blood (Fig. 6E through G).

Additionally, we established the receiver operator characteristic curves of microbial reads number in BALF for distinguishing co-detection of each pathogen in blood and not. As demonstrated in Fig. 6D, bacterial reads in BALF performed better, with an AUC of 0.721. Besides, the optimal cutoff value, determined by maximizing the Youden index, was found to be 2,973 RPM for bacteria (Table 2). Also, bacterial reads in BALF that were detected by PC-mNGS could be applied to predict the co-detection in blood by mNGS, with an AUC of 0.784, and the optimal cutoff value was 3,625 RPM (Fig. 6H; Table 3).

**TABLE 2:** Establishment of the PC-mNGS BALF RPM cutoff value and ROC curve for predicting co-detection in paired blood samples^*a*^

Variants	Cutoff (BALF RPM)	Sensitivity%	1-Specificity%	Youden index maximum	AUC
Bacteria	2,968	54.2	15.0	0.391	0.721
Fungi	138	60.6	33.3	0.273	0.642
Virus	1,423	89.5	68.0	0.214	0.544
Total microorganisms	1,319	52.9	21.3	0.316	0.692

^*a*^ A total of 621 pairs of samples were analyzed, and both BALF and blood were sequenced by PC-mNGS. The cutoff values, sensitivity, and 1-specificity corresponding to the Youden index maximum, and AUC of BALF RPM were determined by receiver operating characteristic curves. The microorganisms at species level that were detected in BALF were assigned to the blood co-detected and blood negative groups. AUC, area under the curve; BALF, bronchoalveolar lavage fluid; RPM, reads per million; PC-mNGS, probe-capture metagenomic next-generation sequencing.

**TABLE 3:** Establishment of the PC-mNGS BALF RPM cutoff value and ROC curve for predicting co-detection in paired blood samples using mNGS^*a*^

Variants	Cutoff (BALF RPM)	Sensitivity%	1-Specificity%	Youden index maximum	AUC
Bacteria	3,625	69.7	17.4	0.523	0.784
Total microorganisms	653	75.6	33.1	0.425	0.768

^*a*^ A total of 83 pairs of samples were analyzed, and BALF was sequenced by PC-mNGS while blood was sequenced by mNGS. The cutoff values, sensitivity, and 1-specificity corresponding to the Youden index maximum, and AUC of BALF RPM were determined by receiver operating characteristic curves. The microorganisms at species level that were detected in BALF were assigned to the blood co-detected and blood negative groups. AUC, area under the curve; BALF, bronchoalveolar lavage fluid; RPM, reads per million; PC-mNGS, probe-capture metagenomic next-generation sequencing.

## DISCUSSION

In this study, we introduced a paired comparison of performance in identifying pathogens between PC-mNGS and mNGS. Analysis from 282 clinical samples revealed that PC-mNGS showed a higher positivity rate than mNGS, particularly in blood samples, under the workflow used in this study. Further analysis showed that the enhanced detection rate of PC-mNGS is largely attributable to its superior detection of bacteria, nearly 45% (19/43) of which were missed by mNGS. Additionally, we screened a large number of paired samples (621 pairs of BALF and blood) to evaluate the detective performance of metagenomic sequencing using different sample types. Expectedly, BALF mNGS identified more pathogens than blood, particularly for viruses, the majority of which were only detected in BALF. Moreover, through analyzing microbial reads number, we found that bacterial reads number in BALF mNGS had the potential in predicting blood co-detection.

To our knowledge, this study represents one of the largest multi-sample-type head-to-head evaluations comparing probe-capture mNGS with conventional shotgun mNGS across three major clinical specimen types (BALF, blood, and CSF) and extends our previous findings in sepsis patients (5). Furthermore, the inclusion of 621 paired BALF-blood samples provides, to date, the largest paired-sample analysis examining compartment-specific pathogen detection patterns using probe-capture metagenomics. Hybridization capture techniques provide a strong balance between specificity, multiplexing, and scalability while enabling the efficient enrichment of many targets. Furthermore, they are comparable in size to the current high-throughput NGS and are now standard techniques for addressing a wide range of issues, including the identification of infectious agents, intricate investigation of microbial ecosystems, and the exploration of the causes of genetic diseases (20). The current study showed a significant increase in pathogen detection rate using PC-mNGS among blood samples. Nonetheless, no significant difference was observed among BALF, CSF, and other samples in their detection rates. In contrast to BALF and CSF, blood is a high host background sample, that is, metagenomic sequencing reads that are annotated as human account for a relatively higher proportion (21). In a study of blood metagenomic sequencing from 78 postoperative patients and 10 healthy people, an average of 95.6% of sequencing reads were mapped to the human genome, and less than 1% of sequences were localized to the genomes of specific bacteria, fungi, and viruses (22). Human-to-microbial DNA ratios may be even higher in samples from inflamed and/or infected sites due to the influx of immune cells (23). It is known that the predominance of host nucleic acid over pathogen nucleic acid significantly limits the overall analysis sensitivity of mNGS (24, 25). Hybridization using capture probes constitutes one of the methods for host DNA/RNA depletion (24), which can capture specific target sequences by designed probes (26). Therefore, in this study, PC-mNGS, which combines hybridization using capture probes targeting pathogen sequences and mNGS, remarkably outperformed traditional mNGS among blood samples.

Due to the absence of a microbiological reference standard (conventional microbiological testing, CMT; clinically diagnostic), we applied PPA, NPA, and OPA to investigate the results agreement between two assays, rather than sensitivity and specificity (27). We found that, among different sample types, BALF obtained the highest OPA of 96.30%, with a PPA of 98.44% and an NPA of 88.24%. A previous study which compared the metagenomic and targeted NGS for detection of respiratory pathogens from BALF showed an OPA of 65.6%, with a PPA of 45.9% and an NPA of 85.7% (28). The results from this study suggested the comparable performance between PC-mNGS and mNGS in detecting pathogens among BALF, indicating that mNGS is performing extremely well in terms of pathogen detection performance on these samples. Besides, it was observed that blood samples had the lowest OPA (70.92%) among all sample types. Several samples were only positive by PC-mNGS, leading to the low NPA (54.05%), and also confirmed the advantages of PC-mNGS for blood detection.

Beyond pathogen detection, the comparison of microbial read yield provided additional insight into platform performance. PC-mNGS enriched microbial reads by more than sixfold relative to mNGS. This enrichment is consistent with the mechanism of probe-mediated host DNA depletion, which frees sequencing capacity for pathogen-derived sequences. This interpretation is further supported by the observation that among pathogens co-detected by both methods, PC-mNGS consistently recovered more reads per pathogen for bacteria and fungi, indicating that the enrichment step concentrates sequencing effort on genuine targets.

The observation that certain pathogens were detected exclusively by mNGS while others were detected exclusively by PC-mNGS warrants mechanistic consideration. The exclusive detections by PC-mNGS, predominantly low-abundance bacteria and fungi in blood, are most parsimoniously explained by the probe-capture enrichment step, which selectively concentrates pathogen-derived sequences against the overwhelming background of host nucleic acid in blood (>95% of reads in blood samples typically align to the human genome [5]). These organisms likely represent genuine but low-biomass pathogens that fell below the limit of detection of conventional mNGS. Conversely, the organisms detected exclusively by mNGS (12 bacteria, 7 fungi, 4 viruses in the 282-sample cohort) may reflect competitive hybridization suppression in co-infected samples with high dominant-organism loads, where abundant target sequences may reduce capture efficiency for low-abundance species. These considerations underscore the complementary nature of both methods and suggest that in clinical practice, a tiered approach—using PC-mNGS as a primary screen for high host-background samples while retaining conventional mNGS for broader, unbiased coverage—may optimize diagnostic yield. The high frequency of *E. faecium* detection in blood samples observed in this study is consistent with the evolving local epidemiology in China. In recent years, *E. faecium* has emerged as a leading cause of hospital-acquired bloodstream infections (BSI) in Chinese intensive care units (ICUs), particularly among sepsis patients with pulmonary infections and prior broad-spectrum antibiotic exposure (5, 29). This contrasts with the relatively lower detection of *Staphylococcus aureus*, which may reflect the predominance of Gram-negative bacteria and secondary BSI from respiratory sources in our cohort. These findings align with multiple Chinese studies reporting *E. faecium* as the predominant enterococcal species in critically ill patients, often associated with high multidrug resistance rates (29).

Using a large sample size, we investigated the pathogens’ spectrum differences between BALF and paired blood. We found that the most frequent pathogens that were co-detected in BALF and paired blood were Gram-negative bacteria, predominantly *Klebsiella pneumoniae*, *Acinetobacter baumannii*, and *Pseudomonas aeruginosa*, followed by fungi, such as *Aspergillus fumigatus*, *Pneumocystis jirovecii*, and *Candida albicans*, all of which are well-established causative agents of secondary BSI in the ICU setting (30). On the contrary, RNA viruses represented by SARS-CoV-2 and influenza viruses were only detected in BALF, except for one case of SARS-CoV-2 with high reads in BALF (10,598 RPM) that was co-detected in blood. The pattern of viral detection differed substantially and requires separate mechanistic consideration. It is important to note that viruses, unlike bacteria and fungi, do not cause “BSI” in the conventional microbiological sense. Viral detection in blood reflects either a viremic phase inherent to the virus’s lifecycle or infection of circulating immune cells (e.g., lymphocytes, monocytes)—neither of which constitutes BSI (31, 32). Respiratory RNA viruses (SARS-CoV-2, influenza A H1N1) were almost exclusively detected in BALF, consistent with their primary tropism for respiratory epithelial cells and their limited capacity to achieve systemic viremia in most clinical scenarios (33). The single exception—one SARS-CoV-2 case with unusually high BALF reads (10,598 RPM) that was also blood-positive—likely reflects the documented phenomenon of SARS-CoV-2 RNAemia in severe COVID-19, which is associated with high pulmonary viral burden, endothelial involvement, and adverse outcomes (34).

Noteworthily, except for one case of SARS-CoV-2, the remaining 18 cases of viruses co-detected in blood were DNA viruses, accounting for 18 of the 19 virus-blood co-detection cases, including human adenovirus (8 cases), human parvovirus B19 (6 cases), and human herpesvirus (4 cases). Critically, in 10 of these 18 samples, blood reads equaled or exceeded BALF reads, a pattern incompatible with pulmonary translocation and strongly suggestive of independent hematogenous replication. Human parvovirus B19 is a classic hematotropic virus that replicates in erythroid progenitor cells in bone marrow, achieves high-level viremia during primary infection, and can persist in blood through reactivation (35). Human adenovirus is well recognized for causing disseminated viremia and end-organ disease in immunocompromised patients (36). Human herpesvirus family members, particularly EBV (HHV-4) and HSV-1 (HHV-1), establish latency in circulating B lymphocytes and can reactivate under conditions of immune suppression, resulting in detectable viral DNA in blood without constituting BSI (37, 38). These findings underscore that blood DNA virus detection in our cohort most likely reflects viremic reactivation or hematogenous tropism rather than secondary spread from pulmonary foci.

After analyzing the reads number, we demonstrated that for double-positive pathogens among BALF and blood, bacterial and fungal pathogens exhibited considerably higher reads number in BALF than in blood, which could reflect either true bloodstream involvement or circulating microbial nucleic acids/cell-free microbial DNA (cfDNA) derived from the pulmonary infection site. However, it is important to note that microbial nucleic acid detection in blood does not necessarily indicate true BSI. Given that our blood processing protocol—differential centrifugation, followed by supernatant collection—retains both intact microbial cells and circulating cfDNA, the detected sequences in blood may reflect: (i) viable pathogens that have translocated from the infected lung tissue to the bloodstream, ultimately resulting in secondary BSI; or (ii) circulating cfDNA or extracellular nucleic acids derived from the pulmonary infection site, passively released into the circulation without active bacteremia/fungemia. Primary BSI often does not have a clear primary source, and most BSIs are secondary, which can originate from intravascular catheters, abdominal infections, urinary tract infections, and pneumonia (39). In the setting of an ICU, pneumonia was the most frequent source of BSI, and a higher risk of death was reported when pneumonia was the source of ICU-BSI (30). A retrospective study involving 141 cases of hospital-acquired *Acinetobacter baumannii* bacteremia indicated that patients in the pneumonia-related group exhibited poorer prognoses, increased antibiotic resistance, and higher complication rates compared to those without pneumonia (40). As a result, we tried to construct a diagnostic model using the pathogen reads number in BALF to predict their co-detection in blood. Results showed that bacterial pathogens’ RPM performed well to predict their co-detection in blood, with AUCs of 0.721 and 0.784, and cutoff values of 2,968 and 3,625 RPM. The results indicated that patients with BALF bacterial RPM exceeding the identified thresholds (2,968 RPM by PC-mNGS; 3,625 RPM by mNGS) should prompt heightened clinical vigilance for possible bloodstream involvement, including consideration of blood culture and empirical BSI-targeted therapy, regardless of the precise underlying mechanism. NGS-derived pathogen reads have been previously validated as semi-quantitative surrogates of pathogen burden that correlate with disease trajectory and treatment response (41, 42), further supporting the clinical relevance of our proposed threshold.

Regarding the quantitative interpretation of RPM values reported in this study, it is important to note that the probe-capture enrichment step in PC-mNGS fundamentally alters the composition of sequencing output by selectively concentrating pathogen-derived sequences relative to host reads. As a result, for a given pathogen at equivalent biological abundance, PC-mNGS will systematically yield higher RPM values than conventional mNGS. This enrichment-driven amplification of the microbial signal enhances the detectability of low-abundance pathogens, particularly in high-host-background matrices, such as blood. However, it also means that RPM values generated by PC-mNGS and conventional mNGS are not directly comparable, and platform-specific positivity thresholds must be established independently. Consistent with this, the optimal bacterial RPM cutoff values for predicting bloodstream co-detection differed between PC-mNGS (2,968 RPM, AUC = 0.721) and conventional mNGS (3,625 RPM, AUC = 0.784), reflecting the distinct analytical backgrounds of the two platforms. Accordingly, RPM values in both assays should be interpreted as semi-quantitative surrogates of pathogen burden, with clinical thresholds calibrated to the specific platform and sample type in use.

The strengths of this study include its large sample size, innovative comparisons of paired samples, and meticulous efforts to exploit the clinical potentialities of detected microbial reads number. However, the study has limitations. A central challenge lies in lacking the microbiological results from CMTs and a definite diagnosis, hampering the investigation of diagnostic performance. Therefore, “PC-mNGS-only” detections may reflect higher analytical sensitivity, but they remain unconfirmed without an external gold standard. Additionally, we didn’t collect clinical characteristics of patients, which could be integrated into diagnostic classifications or outcome predictions. Furthermore, since our blood nucleic acid extraction protocol captures both intact microbial cells and cfDNA, blood-positive detections may represent either genuine bloodstream pathogens or circulating microbial cfDNA shed from pulmonary foci. Integration with blood culture results and clinical BSI diagnostic criteria (e.g., SEPSIS-3) will be necessary to discriminate these two entities in future studies.

Despite these limitations, the current study conducted a paired comparison between PC-mNGS and mNGS in detecting pathogens, suggesting a potential advantage of PC-mNGS in identifying pathogens, especially among blood samples for bacteria, with a significantly higher positive rate. In addition, through a large sample size of paired BALF and blood samples, our data enhanced the understanding of microbiological differences between the lung and bloodstream, indicating the potential of BALF pathogens’ reads number in predicting BSI.

## Data Availability

The raw sequence data reported in this paper have been deposited in the Genome Sequence Archive (Genomics, Proteomics, and Bioinformatics 2025) in the National Genomics Data Center (Nucleic Acids Res 2025), China National Center for Bioinformation/Beijing Institute of Genomics, Chinese Academy of Sciences (GSA: CRA038550 and CRA038813) under the BioProject: PRJCA037107 that are publicly accessible at https://ngdc.cncb.ac.cn/search/specific?db=bioproject&q=PRJCA037107.

## SUPPLEMENTAL MATERIAL

Figure S1 spectrum.04057-25-s0001.docx Comparison of pathogen read counts between PC-mNGS and conventional mNGS.

Figure S1 spectrum.04057-25-s0002.tif Image file for Fig. S1.

Tables S1 to S3 spectrum.04057-25-s0003.xlsx Table S1: Clinical reportable pathogen and pathogen used for probe design. Table S2: The list of accession ID of our database. Table S3: Summary of samples and detected species.

ASM does not own the copyrights to Supplemental Material that may be linked to, or accessed through, an article. The authors have granted ASM a non-exclusive, world-wide license to publish the Supplemental Material files. Please contact the corresponding author directly for reuse.
