# Supplementary material for: Comparative evaluation of probe-capture and conventional metagenomic sequencing across multiple clinical sample types, with analysis of paired bronchoalveolar lavage fluid and blood samples
Source: Microbiol Spectr. 2026 Aug 4;14(9):e04057-25. doi: 10.1128/spectrum.04057-25 (PMC13532337; doi:10.1128/spectrum.04057-25)
Supplement: Supplementary file 1 [file spectrum.04057-25-s0001.docx]

Figure S1 | Comparison of pathogen read counts between PC-mNGS and conventional mNGS.

A, Comparison of total microbial read yields between probe-capture metagenomic sequencing (PC-mNGS) and conventional metagenomic sequencing (mNGS) across 282 paired clinical samples. B, Paired comparison of read counts for co-detected species stratified by pathogen category: bacteria, fungi and viruses. Read counts are shown on a log10 scale. C, Paired comparison of pathogen read counts for species detected by both methods in at least 10 sample-pathogen pairs. Pathogens are ordered from left to right by co-detection frequency. Each point represents one co-detected sample-pathogen pair; boxes show the median and interquartile range, and whiskers indicate 1.5 times the interquartile range. Yellow denotes PC-mNGS and orange denotes conventional mNGS. *p* values were calculated using paired Wilcoxon signed-rank tests; ns indicates *p* ≥ 0.05. The number below each label indicates the number of paired detections.
